# Cystic Fibrosis With No Known Cystic Fibrosis Transmembrane Conductance Regulator (CFTR) Mutation and a Negative Newborn Screening Test: A Case Report

**DOI:** 10.7759/cureus.108603

**Published:** 2026-05-10

**Authors:** Rachana Subbanna, Kathleen Martin

**Affiliations:** 1 Pediatrics, West Virginia School of Osteopathic Medicine, Lewisburg, USA

**Keywords:** cftr gene mutation, cystic fibrosis (cf), cystic fibrosis transmembrane conductance regulator (cftr) protein, newborn screen, sweat chloride

## Abstract

Cystic fibrosis (CF) is a progressive, autosomal recessive disease primarily affecting the pulmonary and gastrointestinal systems of afflicted individuals. Cystic fibrosis transmembrane receptor (CFTR) genes encode the CFTR protein, which allows chloride ions to pass from inside the cell to the outside of the cell. CF symptoms, such as thick, sticky mucous, result from CFTR gene dysfunction. Although many mutations in the CFTR gene have been identified, there remains an absence of a comprehensive database encompassing all known and potential mutations contributing to the pathogenesis of CF.

We present a 12-year-old male with recurrent sino-pulmonary infections, asthma exacerbations, and failure to thrive. Despite a negative newborn screening test, CF was suspected. The sweat chloride test is considered the gold standard for confirming CF. This patient’s sweat chloride test showed elevated levels of 63 and 66 mmol/L, respectively. Levels >60 mmol/L are considered consistent with CF. CF genotyping, however, did not uncover a known CFTR mutation.

The purpose of this case report is to highlight the importance of the sweat chloride test in diagnosing CF in the setting of a negative newborn screen and the absence of a known CFTR mutation, to enhance the diagnosis and recognition of CF in pediatric patients, and to ultimately improve patient outcomes.

## Introduction

Cystic fibrosis (CF) is a common autosomal recessive disease involving a mutation of the cystic fibrosis transmembrane receptor (CFTR) gene, which encodes the CFTR protein, allowing chloride ions to pass from inside the cell to the outside of the cell. After chloride ions pass, water follows to thin out the mucus. The CFTR protein carries a key function in the respiratory, digestive, and reproductive systems, including playing a key role in the reabsorption of chloride in sweat glands [1]. This creates the basis of the sweat chloride test, a critical diagnostic test for patients suspected of having CF. According to Stephenson et al., the incidence of CF in the United States has historically been 1:4,000. However, in 2019, the estimated incidence of CF was calculated to be 1:5,130 (95% CI: 1:4,996, 1:5,267), and it has decreased at a rate of 1.5% per year since 1995, even while birth rates have remained stable [2].

According to the West Virginia Department of Health and Human Resources (WVDHHR), all infants born in West Virginia (WV) undergo newborn screening for various serious medical conditions mandated by state code H.B. 2583. Screening for CF was added to the list of mandated conditions on March 3, 2008, and, to date, all infants born in WV are screened for CF between 24 and 48 hours following delivery [3]. The aim of the newborn screening test is to identify CF in neonates prior to the development of signs and symptoms. False-negative newborn screening tests have been discussed in the literature and can lead to significant delays in care for patients with CF.

Newborn screening begins with the determination of immunoreactive trypsinogen (IRT) concentrations from dried blood spots [4]. IRT is an isoform of trypsinogen, which is a precursor protein synthesized and stored in the pancreas, and patients with CF may present with elevated IRT values in the neonatal period, presumably from leakage of protein into circulation after exocrine pancreatic injury [5]. The WVDHHR describes two different approaches for analyzing IRT levels, one being the performance of mutation analysis and another based on persistent elevation of IRT concentrations requiring a second dried blood spot two to three weeks after birth. Sensitivity for both techniques is approximately 95%, while specificity varies by technique: specificity of mutation analysis after initial IRT evaluation is 99.9%, while without mutation analysis, it is 99.5% [4]. Diagnosis of CF can be made if two mutations are identified on IRT analysis testing; however, if only one mutation is identified, then sweat chloride testing must be performed on infants more than one week of age [4]. If the newborn screening is deemed positive, a sweat chloride test is indicated. However, a sweat chloride test may also be indicated if the newborn screening is deemed negative but the clinical presentation is consistent with CF.

Sweat chloride testing is performed on a newborn no earlier than 48 hours after birth, as sweat chloride levels may be elevated immediately following birth. A clinician performs pilocarpine iontophoresis to stimulate the sweat glands on the arm or leg of an infant and collects samples [6]. Currently, newborns with a sweat chloride value greater than 60 mmol/L are diagnosed with CF, a level between 30 and 59 mmol/L is considered equivocal, and a level less than 29 mmol/L is considered normal [6]. For patients with equivocal sweat chloride test levels, genetic analysis should be considered, and three approaches can be applied. First, if the patient has two known CF-causing mutations on different chromosomes, a diagnosis of CF is confirmed. Second, if no known CF-causing mutations are found and the patient presents with a clinical presentation suspicious of CF, then additional testing should be pursued. Finally, for patients with unknown CFTR mutations or mutations of uncertain clinical significance, additional testing should be pursued if clinical symptoms are present [6].

For symptomatic patients who are negative for known CFTR mutations, nasal potential difference (NPD) testing may be performed. NPD testing is used to reflect the passage of ions across the nasal epithelium, in part by measuring CFTR function. To perform NPD testing, Na+ ion transport is blocked by perfusion of ENaC-inhibiting agents such as amiloride [7]. Perfusion of a chloride-free solution induces the movement of chloride ions through CFTR channels, in addition to isoproterenol, which activates the CFTR channel [7]. Finally, ATP is perfused to serve as a marker of the integrity of the nasal epithelium. Ultimately, total chloride conductance is measured and compared to the mean. 

## Case presentation

**Figure 1:**
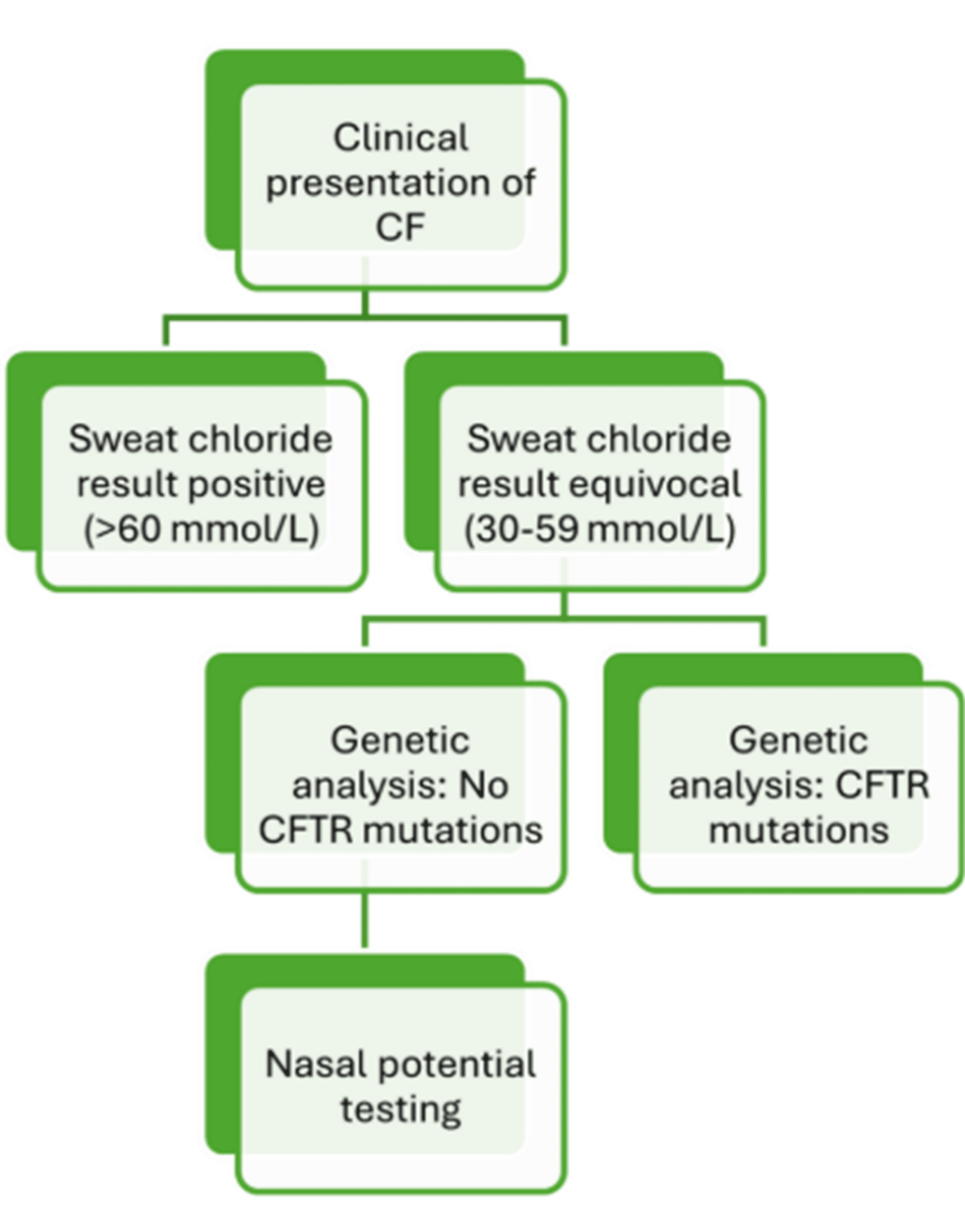
Algorithm for diagnosis of CF with equivocal or positive sweat chloride Source: Adapted from Cystic Fibrosis Foundation [8]. CF: cystic fibrosis; CFTR: cystic fibrosis transmembrane receptor

The purpose of this case report is to evaluate the diagnostic methods of CF discussed above, to emphasize the importance of recognizing the clinical presentation of CF in the diagnostic schema in Figure 1, to enhance the diagnosis and recognition of CF in pediatric patients, and, ultimately, to improve patient outcomes. In this report, we present a case of a 12-year-old male with a negative newborn screening and no known CFTR mutation, who was diagnosed with CF. 

**Figure 2:**
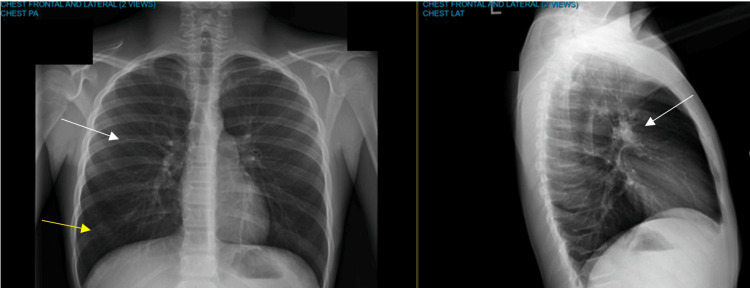
AP and lateral views of peribronchial thickening (white arrows) and the air-trapping phenomenon (yellow arrow)

We present a 12-year-old male child with recurrent sinopulmonary infections starting at two years of age, moderate persistent asthma, and failure to thrive by age 7 years. Neonatal medical history was unremarkable, with a negative newborn screening. The patient was referred to Pediatric Pulmonology at age 7 for suspected CF. A chest X-ray was performed and showed generalized air trapping and peribronchial cuffing, as demonstrated in Figure 2. Pulmonary function testing was interpreted as normal spirometry, with an FEV1 of 105%, FVC of 112%, and no change post-bronchodilator administration. Full CFTR sequencing with duplication/deletion analysis was conducted at Mayo Laboratories, and no CFTR mutations were identified. A sleep study was performed, with results showing mild obstructive sleep apnea and no hypoventilation. Pancreatic enzymes were within normal limits. Two sweat chloride tests were collected (47 and 55 mmol/L, and 53 and 57 mmol/L), which were deemed equivocal. 

The patient was referred to Cincinnati Children’s Hospital Medical Center at age 8, where a third and final sweat chloride test revealed values of 63 and 66 mmol/L, which were considered positive results. NPD testing was performed and demonstrated total chloride conductance one standard deviation below the healthy control mean value. This is considered an intermediate result and may be consistent with CFTR dysfunction. Nasal cells were collected, and culture results are pending. CFTR modulator response results may be obtained in the future. 

## Discussion

The newborn screening for CF has significantly improved its early detection; however, it is not diagnostic and may fail to identify a subset of patients with atypical presentations or less common genetic variants [4,9,10]. The present case demonstrates how a negative newborn screening result does not definitively exclude CF when the clinical presentation is suggestive. Lumertz et al. described children with chronic lung disease who initially had false-negative IRT screening results, emphasizing that reliance solely on newborn screening may delay diagnosis and treatment in symptomatic patients [9]. Similarly, Dunn et al. reported a pediatric patient with a false-negative newborn screening who was later diagnosed with CF following the development of characteristic respiratory manifestations, highlighting the need for ongoing clinical vigilance [10]. Stephenson et al. noted that, while the incidence of CF has slightly decreased in recent years, the disease continues to present with varying phenotypic presentations [2]. In such cases, physicians must rely on a combination of clinical presentation and additional diagnostic tools, such as repeated sweat chloride testing and NPD testing [7,8]. Other immunodeficiencies may mimic CF and must be considered in the differential diagnosis, such as common variable immunodeficiency, primary ciliary dyskinesia, and severe combined immunodeficiency, as these may also present with symptoms of chronic cough, recurrent sinopulmonary infections, and failure to thrive.

Sweat chloride testing remains the diagnostic gold standard for CF if values are greater than 60 mmol/L [6]. However, equivocal results between 30 and 59 mmol/L, as seen in this patient, may complicate the diagnostic process and require repeat testing or referral to specialized CF centers [8]. Previous literature has demonstrated that repeated sweat chloride testing can ultimately yield diagnostic results in patients who initially present with borderline values [6]. NPD testing can also provide important functional evidence of CFTR dysfunction in patients without clearly identifiable mutations, as described by Rowe et al., who demonstrated the utility of this test in evaluating ion transport abnormalities associated with CF [7].

This case highlights the importance of integrating clinical judgment with diagnostic testing when evaluating suspected CF. There is a need for consensus on the diagnosis of CF due to the broad range of clinical symptom presentations and varying diagnostic results. The CF newborn screening test is not a diagnostic test but rather a screening test; therefore, positive screening results must be confirmed with a positive sweat chloride test, which is the gold standard. Negative screening results, however, should not be overlooked, as false negatives can result in delayed diagnosis if clinical signs of the disease are disregarded [9]. Furthermore, false-negative newborn screening results may occur due to laboratory variability, lower IRT levels that fall below screening thresholds, or certain clinical presentations such as meconium ileus [10].

Careful consideration of the diagnostic schema should be applied to patients who present with signs and symptoms of CF. Prompt recognition of the clinical presentation is necessary, as late diagnosis may lead to increased morbidity and mortality, deterioration of digestive and pulmonary function, malabsorption, failure to thrive, and infertility. Patients with delayed diagnosis may experience treatment challenges such as recurrent pulmonary exacerbations requiring hospitalization or frequent antibiotic therapy, chronic airway colonization with resistant organisms, or irreversible pulmonary decline prior to specialty intervention. Early referral to specialized CF centers and the use of advanced diagnostic techniques are essential for patients with persistent symptoms suggestive of CF [8]. This case reinforces the importance of maintaining a high index of suspicion and pursuing comprehensive diagnostic evaluation when clinical findings are consistent with CF despite negative screening results [4,6,7]. 

## Conclusions

The diagnosis of CF can be challenging in patients with negative newborn screening results and other atypical presentations. This case illustrates the importance of integrating clinical findings with repeat diagnostic testing when CF remains part of the clinical picture. Despite an initially negative newborn screen and the absence of identifiable CFTR mutations, persistent symptoms and equivocal diagnostic results warranted further evaluation, ultimately leading to a confirmed diagnosis. Clinicians should maintain a high index of suspicion for CF in patients with recurrent respiratory infections, failure to thrive, or other characteristic features, even when initial screening results are negative. Early recognition and comprehensive diagnostic evaluation are essential to ensure timely management and reduce the long-term complications associated with delayed diagnosis.
